# Genomics for all in the 21st century?

**DOI:** 10.1007/s12687-017-0333-5

**Published:** 2017-09-14

**Authors:** Martina C. Cornel, Vence L. Bonham

**Affiliations:** 1 Department of Clinical Genetics and Amsterdam Public Health Research Institute, Section Community Genetics, VU University Medical Center, BS7, A527, Mail A509 APH, PO Box 7057, 1007 MB Amsterdam, The Netherlands; 2 Division of Intramural Research, Social and Behavioral Research Branch, National Institutes of Health, National Human Genome Research Institute, Bethesda, MD USA

**Keywords:** Genomics, Health equity, Diversity, Health care disparities, Community engagement

## Abstract

As the field of genomics enters the second decade after the completion of the International Human Genome Project, human genomics research is still far from reflective of the ancestral diversity found in global populations. This special issue of the *Journal of Community Genetics* brings together a global perspective on the need for researchers and health care professionals to support achievable milestones that will enhance global ancestral diversity in genomic research for the 21st century, and integrate the resulting knowledge into health care that benefits everyone. As the publications in this special issue illustrate, this will require focused community engagement, including often overlooked isolated populations, as well as meaningful integration of genomics and health services across the global landscape. With the advancement of sequencing technology and reduction in the cost, the time has come to address critical barriers.

As genomics research enters the second decade after the completion of the International Human Genome Project, human genomics research is still not fully reflective of the ancestral diversity found in global populations (Bustamante et al. 2011; Popejoy and Fullerton 2016). As we advance our understanding of the interaction between gene expression and disease, and given that genomic information has the potential to play a key role in the future of health care, the current lack of inclusion of diverse ancestral populations in genomics research creates a risk of exacerbating existing health care disparities (Manrai et al. 2016; Cornel 2017) and limiting our scientific knowledge of worldwide genetic variation. This is both a scientific and health equity challenge to the global scientific community. What international efforts are needed to increase underrepresented populations within genomics research? How do we ensure that all populations benefit from the promise of genomics?

Health care benefits of genomics have largely been studied in diseases in European ancestral populations only. Even in countries with significant investment in genomic research, some minority communities have been largely left out. Consequently, these populations may not benefit from these genomic advances. Furthermore, genetic services tend to benefit patients and consumers from higher socio-economic backgrounds. Access is codetermined by awareness, education, and income.

The scientific community recognizes that the advancement of the field requires a change. Recently, a number of countries have established population-based genomic, environmental, and behavioral studies that aim to recruit a large number of participants for genomic sequencing, for example, the Precision Medicine Initiative All of Us Research Program in the USA (https://allofus.nih.gov/) and the Genomics England 100,000 Genomes Project (https://www.genomicsengland.co.uk/) in the UK. It is essential that these and future population-based studies recognize the scientific need for inclusion of diverse ancestral populations and use them as a platform for greater community engagement in genomics.

The *Journal of Community Genetics* in its aims and scope states that the journal is “an international focal point for research in the ever-expanding field of community genetics, the art and science of applying medical genetics to human communities for the benefit of their individuals.” Its intention is to serve as a forum for community genetics worldwide, with a focus on low- and middle-income countries. The focus on underserved populations has been explicit in several earlier contributions in the journal, for instance in the special issue on the “Genetic testing in emerging economies (GenTEE)” project (Nippert 2013). The experts chosen were involved in activities to develop genetic services, highlighting network projects in Argentina, Egypt, and South Africa, often starting from needs assessment. This approach starts from knowledge that is available. What is needed now is recruitment and community engagement to enable the understanding of gene variants in all populations, and to integrate this into health care.

In the current issue, contributors introduce strategies to improving recruitment and retention of participants from underrepresented populations, and advancing integration into health care. Broadly, they address equality of access, reducing stigmatization and developing appropriate patient information on recruitment and during the study. Bentley, Callier, and Rotimi present a rationale for conducting genomic research in ancestral diverse populations (2017). They contend that global collaborative efforts will be necessary to ensure that genomic research benefits all. They state the inclusion of diverse populations in genomic research “is not just the right thing to do for reasons of equity, it is a scientific imperative.” They provide a persuasive case why including diverse populations in genomic research can help to facilitate new understanding of human biology important for clinical practice and public health.

Sirisena and Dissanayake address factors which contribute to the global inequities in the benefits of genomics. They present the case that a commitment to support research will be needed for developing nations to fully benefit from the genomic revolution. “By realizing the importance of genomic research and its applications for health, drugs and food security, governmental policies should prioritize research funding for genomics” (Sirisena and Dissanayake 2017). McElfish and colleagues report a model of community-based participatory research (CBPR) to collaboratively conduct genetic research related to cancer, diabetes, birth defects, and perinatal outcomes with a Marshallese community (McElfish et al. 2017). Members of the community were involved in prioritization of the research topics, and an interprofessional team began working with the community on this priority. As a result, a free clinic to treat uninsured Marshallese with diabetes was implemented. Landry et al. (2017) investigated customers of direct-to-consumers (DTC) genetic testing companies and found few differences between white and minority customers. Asian customers reported “limited information about their family health history” as a very important reason to pursue DTC genetic testing.

Minority and indigenous populations have historically had poor access to genetic services, including cancer services (Mathew et al. 2017, Van der Giessen et al. 2017). Different factors may play a role, such as genomic variation which may make the interpretation of results more difficult in non-Western populations and the lack of awareness among health care professionals of genetic factors contributing to diseases in diverse populations. Hemoglobinopathies are genetic diseases that are among the key causes of concern, especially due to their significant impact especially in parts of the world where health systems are developing. Robinson reports on an international initiative that uses hemoglobinopathies as an entry point to promote the use of genomic techniques to support more effective diagnosis, treatment, and prevention (Robinson 2017).

Two articles in this special issue describe how a founder effect can lead to different prevalence of diseases, with negative downstream effects for health services and health outcomes (Kääriäinen et al. 2017, Mathijssen et al. 2017). In Finland, for instance, phenylketonuria (PKU) is extremely rare; therefore, neonatal screening was not initiated decades ago. Other countries used their PKU programs to further develop neonatal screening, but in Finland, it developed only more recently. In Dutch founder populations, certain autosomal recessive disorders are more frequent, but carrier screening panels typically do not include these (Mathijssen et al. 2017). An example of limited awareness is found in the paper by Van den Heuvel et al. 2017): glucose-6-phosphate dehydrogenase (G6PD) deficiency is rare in the Dutch majority population. However, this case study of a negative reaction to fava beans in the diet of asylum seekers illustrates the importance of practical knowledge of genetic variation in health workers. Ironically, medical textbooks tend to describe G6PD deficiency as common knowledge.

To better understand the needs of middle- and low-income countries, a needs assessment may first of all investigate the frequency of disorders and the extent to which measures for prevention have been implemented (Christianson et al. 2013). In order to be able to interpret the frequency of variants, their pathogenicity, and the genotype-phenotype correlation, open databases of genomic information need to include data from all people around the world. In an age where we take the right of all people to profit from science seriously (http://genomicsandhealth.org/), we must move beyond the genomes of the average research participant of Western descent. How health services and genomic testing are related in developing countries needs further exploration. While some diagnostics are currently far too expensive to allow for global accessibility, alternatives may be available that are more suitable when resources are limited.

To fully tackle the problem of lack of genetic diversity in genomic research, the scientific community must define what is meant by achieving the goal of genetic diversity. We argue that genetic diversity requires establishing the commitment of the global genomic research community to support achievable milestones to increase the global ancestral diversity in genomic research for the 21st century. We must take steps, so the research reflects the ancestral fabric of genetic diversity of our world. This will not happen without collaboration and partnership with communities, governments, and scientists across the world. The current approaches to enhance the representation of diverse populations in genomic research are not sufficient to effect the change needed. International organizations and funding agencies working with local communities and researchers across the globe must make ancestral diversity in research initiatives a priority. An international plan as ambitious as the International Human Genome Project is needed to accomplish this goal. With the advancement in sequencing technology and reduction in the cost, the time is now to implement such a plan. The critical barriers today include the lack of international frameworks to harmonize efforts, global research funding, trained scientists from the underrepresented geographic communities, common international data sharing policies, and sustained community engagement and support.

Without the partnership of diverse communities, such a plan will not be successful. We must expand the current dialog to increase the representation of diverse ancestral populations in genomics research at the same time addressing the equitable integration of genomic medicine into health systems and health care delivery to embrace the needs of all.
